# The community pharmacy model for colorectal cancer screening: policy insights from Italy

**DOI:** 10.1093/eurpub/ckac130.111

**Published:** 2022-10-25

**Authors:** S Deandrea, PG Della Valle, F Battisti, P Mantellini, W Mantovani, E Narne, A Odone, C Senore, P Brusa, R Calsolaro

**Affiliations:** 1 Pavia Health Protection Agency, Pavia, Italy; 2 University of Pavia, Pavia, Italy; 3 Istituto per lo Studio, Prevenzione e la Rete Oncologica, Firenze, Italy; 4 Azienda Provinciale per i Servizi Sanitari di Trento, Trento, Italy; 5 Azienda Zero, Padua, Italy; 6 AOU Città della Salute e della Scienza di Torino, Turin, Italy; 7 Università degli Studi di Torino, Turin, Italy

## Abstract

**Background:**

Despite its effectiveness, compliance to colorectal cancer (CRC) screening remains low. Different strategies to improve the adherence were identified, such as the involvement of new stakeholders as the community pharmacists. In Italy a recent national project fuelled the development of this strategy, scaling-up the collaboration between screening programmes and pharmacies formerly at the local level up to the national level.

**Methods:**

The regional representatives of the CRC screening programmes provided to the National Screening Monitoring Centre the agreements arranged between the Regions/Autonomous provinces and their respective pharmacy owners representatives. The agreement decrees were analysed describing the fecal occult blood test pathway (e.g. kit supply and delivery) and supplementary activities provided by the pharmacies together with the CRC screening kit delivery, such as health promotion ones.

**Results:**

Information was received from 18 Regions and Autonomous provinces (86% of the total). Regarding the economic compensation, the amount of money paid for each kit varies a lot, with a range from 0 to 18 EUR. The number of process phases covered by the agreements ranged from a maximum of 16 (out of 18) to a minimum of 0. The processes that were included most often were the supply of the kit, the delivery of the kit, and education/awareness of CRC screening (68.8%), followed by sample transfer to the laboratory, test tube tracing and counselling (62.5%). Among the processes less covered there were the warehouse management and awareness of other healthcare initiatives (12.5%), and only in one case a supplementary agreement on delivery of preparation for intestinal cleansing was included.

**Conclusions:**

The arrangements between pharmacies and CRC screening programmes in Italy are very diverse and unique model is missing. Collaboration between programs and pharmacies is promising and quality standards of the service should be set at international level.

**Key messages:**

- Collaboration between organized colorectal cancer screening programmes and community pharmacies can improve the quality of the service and adherence to screening.
- Quality standards of the pharmacy service in colorectal cancer screening should be set at international level.

